# Comparative Analysis of the Transcriptomic Response to Cisplatin in Drug-Sensitive and Drug-Resistant Testicular Germ Cell Tumors

**DOI:** 10.3390/cancers18040575

**Published:** 2026-02-10

**Authors:** Mehwish Wahid Khan, Doha Shokry, Raya I. Boyd, Ratnakar Singh, Michael J. Spinella

**Affiliations:** 1 Department of Comparative Biosciences, University of Illinois Urbana-Champaign, Urbana, IL 61802, USA; mehwish.wahid@nu.edu.pk (M.W.K.); dshokry@illinois.edu (D.S.); rayaib2@illinois.edu (R.I.B.); 2 Cancer Center of Illinois, University of Illinois Urbana-Champaign, Urbana, IL 61801, USA; 3 Carl R. Woese Institute for Genomic Biology, University of Illinois Urbana-Champaign, Urbana, IL 61801, USA

**Keywords:** testicular cancer, testicular germ cell tumor, cisplatin, chemotherapy resistance, transcriptomics

## Abstract

**Background/Objectives**: Testicular germ cell tumors (TGCTs) are uniquely curable with cisplatin-based therapies even when widely metastatic; however, cisplatin resistance does occur, resulting in very poor prognosis. The mechanisms to explain TGCT hypersensitivity to cisplatin and mechanisms of resistance are not well-understood. **Methods**: The global transcriptional response to acute cisplatin treatment (24 h after a 6 h pulse of cisplatin) was assessed in three parental embryonal carcinoma TGCT cells lines compared to multiple isogenic, stable, cisplatin-resistant clonal lines from these parental cells. **Results**: Cisplatin treatment of parental cells consistently showed a more robust overall transcriptional response to cisplatin compared to their cisplatin-resistant cellular counterparts for a common set of genes and pathways including the upregulation of genes associated with histone modifications and p53, EMT, and KRAS signaling and the downregulation of genes normally upregulated by MYC. Focusing on genes exclusively altered in parental cells revealed upregulated genes known to be induced by p53 and downregulated by MYC and the transferrin receptor, TFRC1. Several of these p53/MYC/TFRC1 targets were associated with a higher instance of disease-free survival in a cohort of TGCT patients. **Conclusions**: Cisplatin resistance in TGCT cells is associated with a diminished alteration in cisplatin-responsive genes, especially genes known to be regulated by p53, MYC and TFRC1, that may be linked to cisplatin hypersensitivity and survival in TGCTs.

## Simple Summary

Testicular cancer is one of the few advanced solid tumors that can be cured with chemotherapy, specifically cisplatin-based therapy. The reasons for this, if better understood, could offer clues to better treat other more chemotherapy-refractory cancers and those rare testicular cancer patients that are resistant to cisplatin. This research uncovered differences in how cisplatin-sensitive and -resistant testicular cancer cells globally respond to cisplatin transcriptionally that may explain and contribute to our understanding of testicular cancer curability and acquired cisplatin resistance.

## 1. Introduction

Chemotherapy is a primary modality for treating various malignancies. However, its clinical efficacy is frequently compromised by the development of resistance. Chemoresistance, whether intrinsic or acquired, represents a major challenge in cancer therapy and is a leading cause of treatment failure and disease relapse. Tumor cells can resist chemotherapeutic agents through multiple mechanisms, including increased drug efflux, altered drug metabolism, mutations in drug targets, enhanced DNA repair capacity, and inhibition of apoptosis [1,2]. Furthermore, the tumor microenvironment, cancer stem cell populations, and epigenetic modifications play pivotal roles in promoting adaptive resistance [3].

Testicular germ cell tumors (TGCTs) are the most common malignancies in men aged 15–45 and, prior to the introduction of cisplatin-based regimens in the 1970s, were a highly lethal disease in the metastatic setting [4,5]. Chemotherapy has dramatically improved survival outcomes in patients with TGCTs, which is one of the most curable metastatic solid malignancies. The introduction of cisplatin-based regimens has led to cure rates exceeding 90% [6,7]. However, a subset of patients develop resistance to chemotherapy, resulting in refractory or relapsed disease with limited therapeutic options and poor prognosis [8]. The mechanisms underlying the hypersensitivity of TGCTs to cisplatin and mechanisms of chemoresistance are likely multifactorial and remain incompletely understood [9].

Our previous studies have elucidated a significant role of epigenetic regulation, mediated by DNA and histone methylation, in determining chemosensitivity or resistance in TGCTs. This included finding that basal repression of the polycomb pathway and basal DNA hypermethylation were associated with cisplatin resistance in isogenic cisplatin-resistant TGCT cell models and that pharmacologic and genetic targeting of these pathways can restore cisplatin sensitivity to these cisplatin-resistant cells in vivo [10,11,12,13]. In order to assess mechanisms downstream of basal epigenetic alterations associated with cisplatin resistance in TGCTs, we compared the acute transcriptional response to cisplatin in these cisplatin-resistant cell models compared to parental cells. Cisplatin resistance was associated with a diminished alteration in the expression of cisplatin-responsive genes, especially in genes known to be regulated by p53, MYC, and the transferrin receptor, TFRC1, and the expression of several of these genes was associated with TGCT patient survival.

## 2. Materials and Methods

### 2.1. Cell Culture and Drug Treatment

Cell culture was performed using DMEM (Corning) media supplemented with 10% FBS (GeminiBio, West Sacramento, CA, USA), 1× antibiotic–antimycotic (Corning, Corning, NJ, USA), and 2 mM L-Glutamine (Corning). The human TGCT embryonal carcinoma (EC) cell lines NT2/D1, 833K, and 2102EP were obtained from ATCC. The process for generating cisplatin-resistant cells has been previously described [12]. In brief, to create cisplatin-resistant cells, the parental cells were exposed to stepwise doses of cisplatin (Sigma, St. Loius, MO, USA) ranging from 0.5 µM to 10 µM for 3 h daily over a period of 5 days, after which they were allowed to recover [12]. The cells were then cloned and shown to maintain their cisplatin resistance in cisplatin-free media for up to 5 months. Each independent cisplatin-resistant line was derived from a unique batch of cisplatin-selected cells. NT2/D1, 833K and 2102EP are wild-type for p53. Detailed mutational analysis of the resistant lines has not been performed.

Prior to any experiments, both the parental and cisplatin-resistant cells were re-evaluated for cisplatin sensitivity using a cell viability assay to confirm cisplatin resistance. For RNA extraction, parental and cisplatin-resistant clonal cell lines were treated with 0.5 µM cisplatin for 6 h. Samples that were cisplatin-treated and untreated were collected 24 h after cisplatin treatment for RNA extraction.

### 2.2. RNA Extraction, RNA Sequencing and Gene Set Enrichment Analysis

RNA was extracted from untreated and cisplatin-treated cells in biological triplicate and RNA sequencing was performed at Carver Biotechnology Center, University of Illinois at Urbana-Champaign, as previously described [10,11,12]. RNA-Seq libraries were prepared and analyzed as previously described using the limma package [10,11,12,14]. The genes with a fold change greater than or equal to 1.3 and an FDR less than 0.01 were considered differentially expressed genes (DEGs) [10]. For each cell line, gene expression was compared between cisplatin treatment and vehicle (PBS) control treatment. The RNA-seq datasets from this study are available at GEO, accession number GSE317098. Separate principal component analysis (PCA) was performed for each cell line set to assess the degree of transcriptional similarity among different resistant cells, as described [10,11,12]. The top 5000 most variable genes (based on variance across all samples) were selected for PCA.

The gene set enrichment analysis (GSEA) was performed with the GSEA tool with a maximum gene set size of 500 and a minimum gene set size of 15 [15]. The number of permutations was 1000. ClusterProfiler package version 4.14.3 was utilized to identify significant gene set overlap between genes altered exclusively in parental cells alone and C2 gene sets from the MSigDB database and the readout for this analysis is the *p* value [16].

### 2.3. Disease-Free Survival Analysis

Survival analysis was performed using TCGA clinical and molecular data from the cBioPortal for Cancer Genomics. Specifically, the Non-Seminomatous Germ Cell Tumor (TCGA GDC, 2025) dataset. After combining the clinical and expression datasets, 104 samples were used for survival analysis. Patient stratification into high- and low-expression groups was performed using the optimal cut-off value for gene expression determined by the maximally selected rank statistics using the survminer R package, version 0.5.1. Kaplan–Meier survival curves were then estimated using the survfit function, and survival differences were assessed using the log-rank test.

## 3. Results

### 3.1. Cisplatin-Resistant Cells Have a Less Robust Transcriptional Response to Cisplatin Compared to Parental Cells

We previously generated a series of seven independent cisplatin-resistant cell lines from three parental embryonal carcinoma cells lines [12]. These cisplatin-resistant cells are isogenic to their parental counterparts and stably cisplatin-resistant for as long as 5 months without the need for continued cisplatin exposure. Prior studies uncovered that these lines all had a basal repression of the polycomb pathway and that a subset of the lines also had basally hypermethylated DNA, suggesting that epigenetic remodeling occurred [11,12]. Further targeting DNA methylation and the polycomb pathway altered cisplatin sensitivity to TGCT cells in vitro and in vivo [10,11,12,13].

In order to assess the ramifications of how epigenetic remodeling leads to cisplatin resistance, the acute transcriptional responses to cisplatin in parental and their isogenic cisplatin-resistant counterparts were compared. For this study 2012EP, NT2/D1 and 833K parental cells and cisplatin-resistant derivatives of these cells were exposed to 0.5 µM cisplatin for 6 h, and RNA was harvested 24 h later for RNA-seq transcriptomic profiling and differential gene expression analysis. This dosing protocol was chosen since prior studies determined that robust transcriptional changes occur in parental cells by 24 h post cisplatin treatment, while apoptosis and cell death require 24–48 additional hours post cisplatin treatment to occur [17].

**Figure 1:**
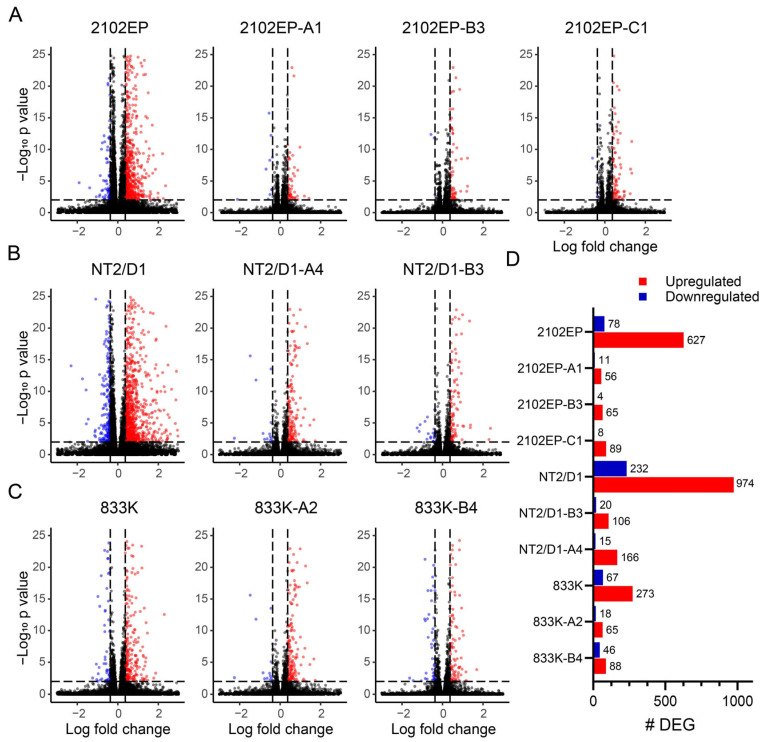
Cisplatin-resistant TGCT cells have a less robust transcriptional response to cisplatin compared to parental cells. (**A**) Volcano plots of the cisplatin response of 2102EP parental and three independently derived cisplatin-resistant cell derivatives. (**B**) Volcano plots of the cisplatin response of NT2/D1 parental and two independently derived cisplatin-resistant cell derivatives. (**C**) Volcano plots of the cisplatin response of 833K parental and two independently derived cisplatin-resistant cell derivatives. (**D**) Number of genes upregulated and downregulated (>1.3 fold change and an FDR < 0.01) after cisplatin treatment in indicated parental and resistant cells.

The transcriptomic response was more robust in all three parental cells after cisplatin treatment compared to their respective acquired cisplatin-resistant cells (Figure 1). The number of genes with altered expression after cisplatin treatment ranged from 340 to 1206 in parental cells and 67 to 181 in cisplatin-resistant lines. A complete list of differentially expressed genes after cisplatin treatment in parental and acquired cisplatin-resistant cells is provided in Supplementary Table S1. Furthermore, we performed principal component analysis which showed good consistency in the biological replicates and that each cell line, whether it is treated or untreated, tends to group separately (Supplemental Figure S1).

### 3.2. Transcriptomic Analysis of Cisplatin-Upregulated Genes Reveals More Robust Enrichment of Genes Related to p53 Signaling, the IR/UV-Based DNA Damage Response, and Epithelial-to-Mesenchymal Transition in Parental Cells Compared to Cisplatin-Resistant Cells

**Figure 2:**
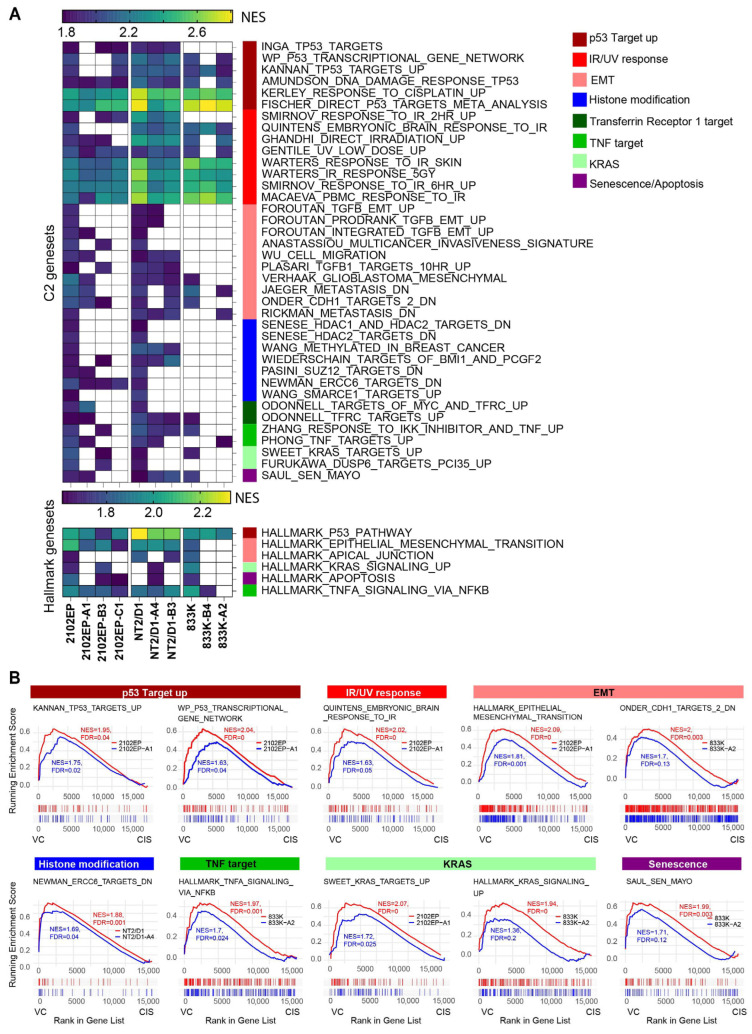
Cisplatin preferentially induces genes associated with activation of p53, DNA damage and apoptosis, and repression of MYC and the transferrin receptor, TFRC1, in parental cells compared to cisplatin-resistant cells. (**A**) Gene set enrichment analysis of cisplatin-upregulated genes reveals more robust pathway enrichment in parental cell lines, including p53 signaling, IR/UV-based DNA damage response, TFRC1/MYC, and epithelial-to-mesenchymal transition signatures. The scale refers to NES. (**B**) Representative gene set enrichment plots. NES, normalized enrichment score; FDR, false discovery rate.

To assess which pathways were altered with cisplatin in cisplatin-sensitive and -resistant cells, gene set enrichment analysis (GSEA) was performed. GSEA of C2-curated gene sets (7561 gene sets) and hallmark gene sets (50 gene sets) revealed that multiple pathways were affected by cisplatin treatment in both parental and acquired cisplatin-resistant cell lines with a normalized enrichment score (NES) of at least 1.8 and an FDR of less than 0.01 (Supplementary Tables S2 and S3). Pathways were selected if enriched in at least two out of three parental cells or in four out of seven resistant cells. To reduce redundancy, the PID, KEGG, and REACTOME gene sets were excluded from the C2 gene set analysis (complete list in Supplementary Table S2). All the gene sets that passed these criteria were further categorized into groups based on their function or phenotype. We and others have previously established that there is a p53-dominant transcriptional response to cisplatin in TGCT cells [18,19,20]. Thus, as expected, many gene sets related to p53 signaling, as well as IR/UV-based DNA damage response, were upregulated following cisplatin treatment in both parental and the majority of resistant cell lines. These gene sets were less enriched in cisplatin-resistant cells (lower NES) compared to their respective parental cells (Figure 2). Similarly, many epithelial–mesenchymal transition gene sets were preferentially enriched in parental cell lines after cisplatin treatment. Chromatin remodeling gene sets, which include histone and DNA modifications, were more enriched in 2102EP and NT2/D1 parental cells in response to cisplatin compared to resistant cells. Interestingly, after cisplatin treatment, the parental cells also had preferential upregulation of genes normally repressed by TFRC1 and MYC. TFRC1 encodes for the transferrin receptor and has been associated with p53 and MYC signaling [21,22]. TFRC1 deregulation has been associated with cisplatin resistance in colon cancer cells and iron metabolism in general has been associated with cisplatin sensitivity in various cancer settings [22,23,24,25]. Additionally, cisplatin-sensitive parental cells had preferential upregulation of apoptotic or senescence pathway genes after cisplatin treatment.

### 3.3. Transcriptomic Analysis of Cisplatin-Downregulated Genes Reveals More Robust Repression of MYC Signaling, RB Targets, and Pluripotent Gene Sets in Parental Cell Lines

**Figure 3:**
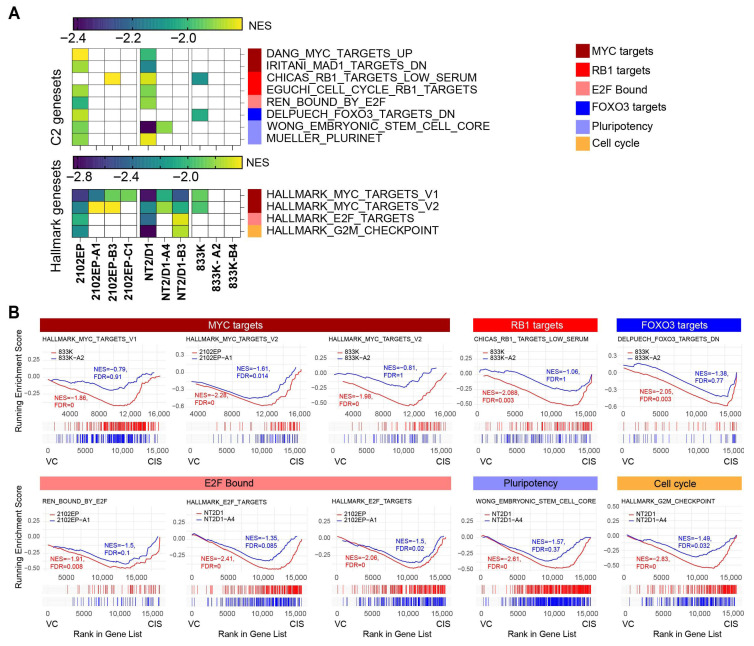
Cisplatin preferentially represses genes associated with MYC/RB/E2F and pluripotency in parental cells compared to cisplatin-resistant cells. (**A**) Gene set enrichment analysis of cisplatin-downregulated genes reveals preferential pathway enrichment for MYC, RB1, E2F, and pluripotency in parental cells. The scale refers to NES. (**B**) Representative gene set enrichment plots. NES, normalized enrichment score; FDR, false discovery rate.

To assess if there are differentially cisplatin-repressed genes between parental and cisplatin-resistant cells, GSEA was also conducted on genes downregulated following cisplatin treatment in both parental and cisplatin-resistant cells. The cut-offs used for NES and FDR, 1.8 and 0.01, respectively, were again used and the same criteria for gene set inclusion, occurring in at least two parental cell lines and four resistant cell lines were used. Several gene sets related to MYC/RB/E2F signaling were preferentially downregulated by cisplatin treatment in parental cells compared to cisplatin-resistant cells (Figure 3, Supplementary Tables S4 and S5). This confirms that cisplatin treatment represses MYC activity by complementing the finding that genes normally repressed by MYC are preferentially upregulated by cisplatin in parental cells (Figure 2). In 2102EP and 833K cell lines, FOXO3 target genes decreased after cisplatin treatment only in parental cell lines. Additionally, cisplatin treatment reduced pluripotency genes and genes related to the G2M checkpoint more prominently in the parental cells after cisplatin treatment.

### 3.4. Analysis of Genes with Uniquely Higher or Lower Expression with Cispatin in Parental Cells

**Figure 4:**
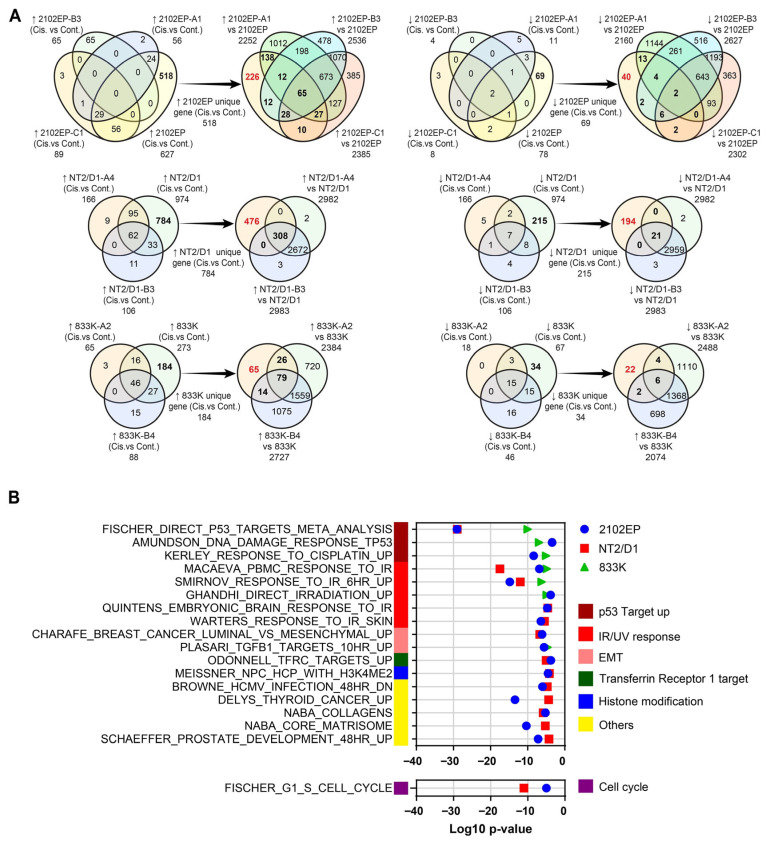
Analysis of genes with uniquely higher or lower expression upon cisplatin treatment of parental cells. (**A**) Strategy to identify genes with uniquely higher or lower expression after cisplatin treatment of parental cells. Bolded numbers in black are genes induced or repressed by cisplatin only in parental cells. Bolded numbers in red are genes induced or repressed by cisplatin only in parental cells and not basally induced or repressed in resistant cells (see text for details). (**B**) Gene set enrichment analysis of genes induced or repressed by cisplatin only in parental cells and not basally induced or repressed in resistant cells (bolded genes in red in (**A**)).

Transcriptomic analysis by GSEA showed that more genes had altered expression in parental cell lines compared to cisplatin-resistant cells after cisplatin treatment. A closer examination revealed that a subset of genes induced by cisplatin in parental cells were basally upregulated in cisplatin-resistant cell lines compared to parental cells. In an attempt to minimize the impact of these basally induced cisplatin-responsive genes in cisplatin-resistant cells in our analysis, they were eliminated in the following manner. A Venn diagram analysis was first performed on genes induced by cisplatin at least 1.3 fold with an FDR less than 0.01 for each parent and that parent’s isogenic cisplatin-resistant cell lines (i.e., the genes in Figure 1D). This resulted in 518, 784 and 184 genes exclusively upregulated by cisplatin in parental 2102EP, NT2/D1 and 833K cells, respectively, compared to their resistant cell counterparts (Figure 4A). Then, further Venn analysis was performed with these genes to remove any genes that were basally upregulated more than 1.3 fold with an FDR of less than 0.01 in resistant cells compared to their respective parent, which left 226, 476, and 65 genes with uniquely higher expression in cisplatin-treated parental cells 2012EP, NT2/D1, and 833K cells, respectively (Figure 4A). Although the phenomena of cisplatin-repressed genes being basally repressed in resistant cells was much less pronounced, a similar analysis was performed for cisplatin-repressed genes (Figure 4A).

To further explore the mechanism of cisplatin response, GSEA was then performed on the genes with uniquely higher or lower expression in parental cells using 7561 curated gene sets. This analysis revealed that gene sets associated with the activation of p53, IR/UV and EMT remained induced exclusively by cisplatin in parental cells, and genes associated with repression of TFRC1/MYC also remained induced exclusively by cisplatin in parental cells (Figure 4B). Among downregulated genes, only G1-S cell cycle genes were enriched exclusively in parental cell lines through this analysis (Figure 4B and Supplementary Table S6).

### 3.5. p53/TFRC1/MYC Target Genes Are Associated with Disease-Free Survival in TGCT Patients

**Figure 5:**
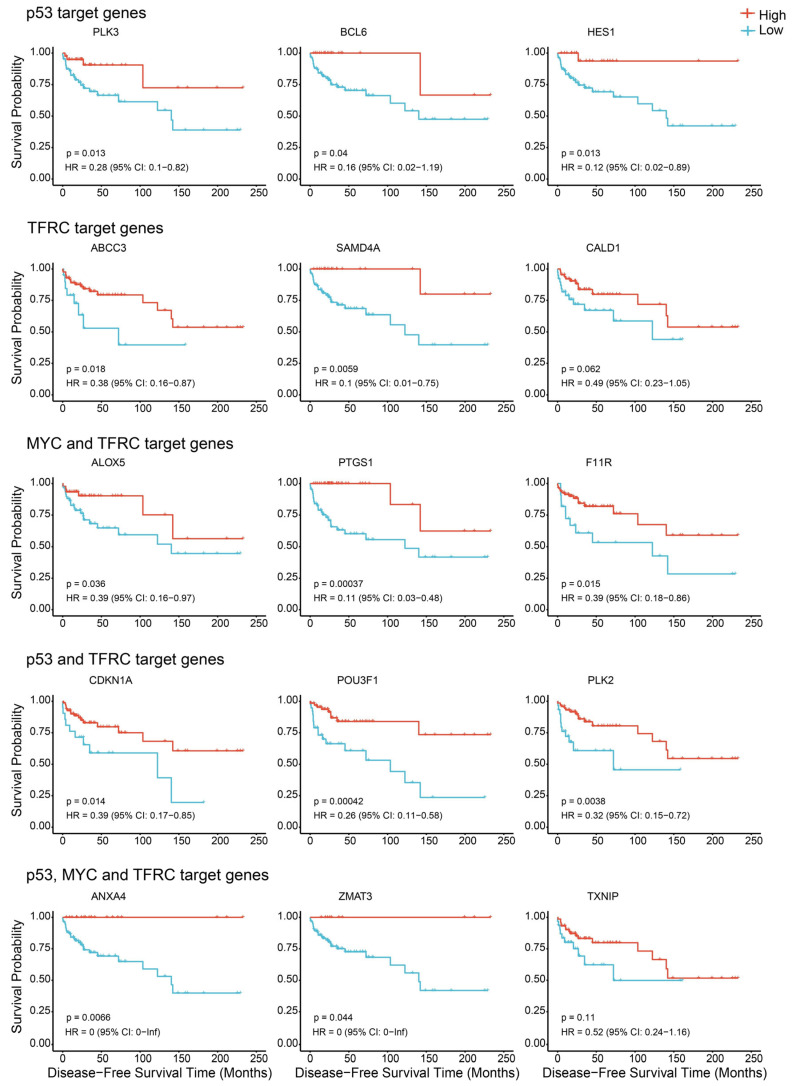
p53/TFRC1/MYC target genes are associated with disease-free survival in TGCT patients. High expression of a partially overlapping subset of genes associated with p53 activation and TFRC1 and MYC repression were associated with increased disease-free survival in TGCT patients as assessed by Kaplan–Meier log-rank tests. HR, hazard ratio; CI, confidence interval.

After cisplatin treatment, parental cells showed preferential upregulation of genes normally repressed by TFRC1 and MYC and upregulation of genes normally activated by p53. Since TFRC1 has been associated with p53 and MYC signaling and its deregulation and iron deregulation in general has been associated with cisplatin efficacy in other cell contexts, the correlation between these targets and disease-free survival in a cohort of TGCT patients was assessed [21,22,23,24,25]. As shown in Figure 5, a subset of genes associated with p53 activation and TFRC1 and MYC repression were associated with increased disease-free survival in TGCT patients, suggesting that these gene may be markers of treatment sensitivity.

## 4. Discussion

Testicular germ cell tumors (TGCTs) are the most common cancer in males aged 15–45 and, when metastatic, were largely a lethal disease before the introduction of cisplatin-based combination therapy in the 1970s [26]. Today TGCTs are a paradigm of a curable solid malignancy in the metastatic setting, although TGCTs in 20% of patients with metastasis are or become refractory to cisplatin and the majority of these patients die from progressive disease [27]. Hence, there is great interest in better understanding why the majority of TGCTs are hypersensitive to cisplatin and mechanisms of resistance in order to more effectively use cisplatin and other chemotherapies for other solid tumors and cisplatin-resistant TGCTs.

Due to their germ cell origins, etiology, and genomics, TGCTs appear to be especially driven by epigenetic alterations [28,29,30]. For example, in prior work leveraging these isogenic cisplatin-resistant cell models, we showed that DNA hypermethylation and polycomb pathway repression were associated with cisplatin resistance in TGCT cells and that pharmacologic and genetic targeting of these epigenetic alterations can restore and/or increase cisplatin sensitivity in TGCT cells [10,11,12,13]. However, these studies did not address the ramifications and downstream mechanisms that are altered after cisplatin treatment due to this basal epigenetic remodeling.

In this report, we performed a comprehensive and global assessment of the acute transcriptional response to cisplatin in parental and isogenic, cisplatin-resistant, TGCT cell counterparts. Cisplatin treatment consistently showed a more robust overall transcriptional response to cisplatin in parental cells compared to cisplatin-resistant cells. This included upregulation of genes associated with histone modifications and p53, EMT, and KRAS signaling, and the downregulation of genes normally upregulated by MYC. Despite changes in EMT genes, there were no obvious change in the morphology of the cells. Furthermore, we found that a partially overlapping set of genes known to be induced by p53 and downregulated by MYC and the transferrin receptor, TFRC1, were preferentially upregulated in parental cells and the expression of several of these genes were associated with a higher instance of disease-free survival in a TGCT patient cohort.

Like most cancer cell chemoresistance, chemoresistance in TGCT cells is likely multifactorial and could include basic mechanisms like increased drug efflux, altered drug metabolism, mutations in drug targets, enhanced DNA repair capacity, and inhibition of apoptosis [1,31,32]. For example, differences in the amount of cisplatin-induced damage or the rate of DNA repair could also influence the transcriptional response to cisplatin seen here in the resistant cells. We previously reported that the amount of cisplatin-induced adducts is similar between parental and resistant lines, but this does not rule out that DNA repair might occur more rapidly in resistant lines, contributing to the different transcriptional responses [12].

In contrast, there is accumulating evidence that the biology of in utero-derived TGCTs including their formation, hypersensitivity to cisplatin, and cisplatin resistance may be especially driven by epigenetic mechanisms [28,29,30]. This includes a low frequency of driver mutations prior to and after emergence of resistance and the link between TGCTs and aberrant in utero exposures to gonadal hormones and environmental contaminants [33,34,35]. In prior work, we showed that the cisplatin-resistant cell models here have undergone epigenetic remodeling that includes DNA hypermethylation and repression of the polycomb pathway and that DNA hypomethylating agents and polycomb demethylase inhibitors can sensitize TGCTs to cisplatin [10,11,12,13]. Here we show that this epigenic remodeling is associated with a quantitative diminishment of the acute transcriptional response to cisplatin in pathways that are known to mediate apoptosis and cell death, including p53 target genes and genes associated with the DNA damage response, senescence, and apoptosis.

Hence, basal epigenetic remodeling may set the stage for a more restrictive transcriptional response to cisplatin in these stably cisplatin-resistant cells. It would be interesting in future work to assess whether basal methylation changes in the resistant cells positively or negatively correlate with transcriptional differences in the cisplatin target genes seen here. Of note, MDM2 is both a p53 target gene and a negative modulator of p53. Similar to other p53 target genes, MDM2 expression levels were increased with cisplatin preferentially in parental cell lines compared to their resistant derivates. However, there was not a consistent pattern of basal MDM2 expression difference between parental and resistant lines.

Many of the pathways noted here preferentially upregulated by cisplatin in parental cells were related to p53. Work by us and others have shown that there appears to be a hyperactivation of p53 in TGCTs in response to cisplatin that correlates with their cisplatin curability and low rate of p53 mutations in these tumors [18,19,20,33]. Further, we also detected a preferential decrease in pluripotency and MYC target genes in parental cells treated with cisplatin compared to resistant cells. Interestingly, our prior work showed that the DNA hypomethylating agent decitabine, that can restore cisplatin sensitivity to cisplatin-resistant TGCT cells, also induced p53 target genes and repressed pluripotency genes and MYC target genes, thus providing a potential mechanism for decitabine reversal of cisplatin resistance in TGCT cells [17].

One of the more interesting findings from this work is the association of cisplatin resistance with diminished cisplatin induction of genes normally repressed by the transferrin receptors, TFRC1 and MYC. In recent years, altered iron metabolism has been linked to the efficiency of cisplatin toxicity in a number of cancer cell types [21,22,23,24,25]. There have also been mechanistic links between MYC, p53 and TFRC1 signaling. For example, O’Donnell et al. found that TFRC1 is a direct target of MYC and Hou et al. suggested that p53 and TFRC1 functionally interact [21,22]. Interestingly, we found that the expression of a subset of these p53/MYC/TFRC1 responsive genes correlated with disease-free survival of TGCT patients. The precise role of TFRC1 and iron metabolism in TGCT cisplatin hypersensitivity and resistance merits further study.

Our work suggests several potential therapeutic strategies for chemoresistant TGCTs. The most obvious is targeting p53 signaling with more efficient p53 activating strategies; for example, the epigenetic drugs that we have already suggested have the ability to enhance p53 signaling in chemoresistant TGCT cells [10,17]. Another strategy suggested by our findings is the targeting of EMT, MYC, TFRC1 and iron metabolism.

There are clear limitations to our study. The work was performed in a series of embryonal carcinoma cells lines. It is critical to expand studies in cell lines of other TGCT histology, including yolk sac tumor, seminoma, and choriocarcinoma, as well as to confirm our findings in clinical TGCTs. Further detailed mutational analysis of the resistant lines can be performed and potential targets identified here including p53, MDM2, TRFC1 and others require mechanistic biological validation.

## 5. Conclusions

Testicular cancer is one of the few advanced solid tumors that can be cured with chemotherapy, specifically cisplatin-based therapy. The reasons for this, if better understood, could offer clues to better treat other more chemotherapy-refractory cancers and those rare testicular cancer patients that are resistant to chemotherapy. This research uncovered differences in how cisplatin-sensitive and -resistant testicular cancer cells globally respond to cisplatin that may explain and contribute to our understanding of testicular cancer curability and acquired cisplatin resistance.

Cisplatin treatment consistently showed a more robust overall transcriptional response to cisplatin in parental cells compared to cisplatin-resistant cells. This included upregulation of genes associated with histone modifications and p53, EMT, and KRAS signaling and the downregulation of genes normally upregulated by MYC. Furthermore, a partially overlapping set of genes known to be induced by p53 and downregulated by MYC and TFRC1 were preferentially upregulated in parental cells, and the expression of several of these genes was associated with a higher instance of disease-free survival in a TGCT patient cohort.

## Data Availability

The RNA-seq datasets from this study have been submitted to the NCBI Database of GEO Datasets under the accession number GSE317098.

## Supplementary Materials

The following supporting information can be downloaded at https://www.mdpi.com/article/10.3390/cancers18040575/s1, Figure S1: PCA of parent and resistant TGCT cells. Table S1: Complete list of differentially expressed genes after cisplatin treatment in parental and cisplatin-resistant cells. Table S2: Curated gene sets predicted by GSEA from upregulated genes after cisplatin treatment in each cell line. Table S3: Hallmark gene sets predicted by GSEA from upregulated genes after cisplatin treatment in each cell line. Table S4: Curated gene sets predicted by GSEA from downregulated genes after cisplatin treatment in each cell line. Table S5: Hallmark gene sets predicted by GSEA from downregulated genes after cisplatin treatment in each cell line. Table S6: Enrichment analysis of genes with uniquely higher or lower expression upon cisplatin treatment of parental cells.

## Abbreviations

The following abbreviations are used in this manuscript:

TGCT	Testicular germ cell cancer
EC	Embryonal carcinoma
EMT	Epithelial–mesenchymal transition
GSEA	Gene set enrichment analysis
FDR	False Discovery Rate
HR	Hazard ratio
CI	Confidence interval
