# Supplementary figures and images for: Comparative Analysis of the Transcriptomic Response to Cisplatin in Drug-Sensitive and Drug-Resistant Testicular Germ Cell Tumors
Source: Cancers (Basel). 2026 Feb 10;18(4):575. doi: 10.3390/cancers18040575 (PMC12938963; doi:10.3390/cancers18040575)

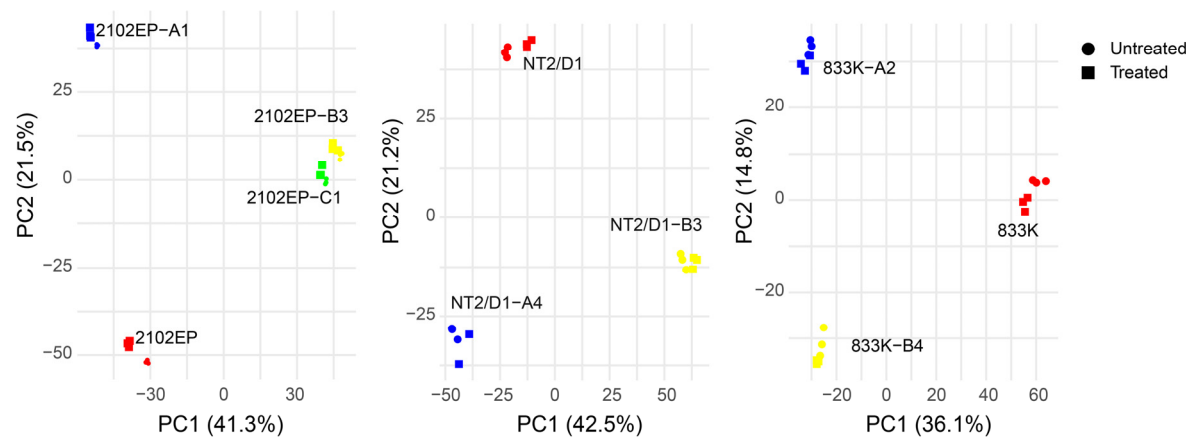

**Figure S1.** PCA of parent and resistant TGCT cells.

Supplement: Supplementary file 1 [file cancers-18-00575-s001.zip › Figure S1.pdf]
